# BacPROTACs targeting Clp protease: a promising strategy for anti-mycobacterial drug discovery

**DOI:** 10.3389/fchem.2024.1358539

**Published:** 2024-01-31

**Authors:** Andressa Francielli Bonjorno, Aline Renata Pavan, Guilherme F. S. Fernandes, Cauê Benito Scarim, Daniele Castagnolo, Jean Leandro Dos Santos

**Affiliations:** ^1^ School of Pharmaceutical Sciences, São Paulo State University (UNESP), Araraquara, Brazil; ^2^ Institute of Chemistry, São Paulo State University (UNESP), Araraquara, Brazil; ^3^ Department of Chemistry, University College London, London, United Kingdom

**Keywords:** *Mycobacterium tuberculosis*, tuberculosis, Bac-PROTAC, ClpC1P1P2, protein degradation, new drugs, drug discovery

## Abstract

Tuberculosis (TB) has claimed more lives over the course of two millennia than any other infectious disease worldwide. In 2021, the World Health Organization (WHO) estimated that 10.6 million people were diagnosed with TB, resulting in the deaths of 1.4 million HIV-negative individuals. The emergence of multidrug-resistant TB (MDR-TB), defined as resistance to at least rifampicin (RIF) and isoniazid (INH), and extensively drug-resistant TB (XDR-TB), poses the primary challenge to overcome in the coming years. We have recently conducted an extensive analysis of investments and research endeavours in the field, with the overarching objective of achieving the established milestone of TB eradication by the year 2030. Over the past several years, there has been notable progress in advancing a multitude of promising compounds, each possessing distinct mechanisms of action, into clinical phases of development. However, it is worth noting that strains of mycobacteria resistant to current antitubercular drugs have already emerged for some of these compounds The exploration of the innovative Proteolytic Target Chimeras (PROTACs) protein degradation approach has emerged as a viable avenue for the discovery of novel antimicrobials. While the ubiquitin system is exclusive to eukaryotic cells, certain bacteria use a similar degradation system that relies on the recognition of phosphorylated arginine residues (pArg) by the ClpC:ClpP (ClpCP) protease, thereby leading to protein degradation. In this opinion article, we have described and analized the advances in the use of PROTACs that leverage bacterial proteolytic machinery (BacPROTACs) to design new antitubercular agents. Scope Statement. The development of novel pharmaceuticals for tuberculosis treatment is deemed urgently necessary due to the emergence of resistant strains. In this context, the introduction of new technologies capable of alleviating the disease and attaining the objectives outlined by the World Health Organization is imperative. Among the innovative strategies, the degradation of proteins that are crucial for the survival of the *bacillus* holds promise for generating new medications, particularly those that are effective at treating latent (non-replicating) *Mycobacterium tuberculosis*. Within this perspective, we present the advancements and obstacles encountered in the exploration of new BacPROTAC compounds, with the intention of encouraging research and illuminating challenges associated with the implementation of BacPROTACs to address to the global tuberculosis crisis.

## 1 Introduction

Tuberculosis (TB) has claimed more lives over the course of two millennia than any other infectious disease worldwide. In 2021, the World Health Organization (WHO) estimated that 10.6 million people were diagnosed with TB, resulting in the deaths of 1.4 million HIV-negative individuals. Additionally, TB claimed the lives of 214,000 HIV-positive individuals. The emergence of multidrug-resistant TB (MDR-TB), defined as resistance to at least rifampicin (RIF) and isoniazid (INH), and extensively drug-resistant TB (XDR-TB), poses the primary challenge to overcome in the coming years. The latest data from WHO regarding drug-resistant TB is alarming, with an estimated 450,000 new cases and 182,000 deaths from MDR- or RIF-resistant TB in 2021, reflecting a 3.1% increase compared to 2020 (WHO, 2022).

We have recently conducted an extensive analysis of investments and research endeavours in the field to assess whether we are on course to achieve the overarching objective of TB eradication by 2030. (Stop TB partnership, 2019; Treatment Action Group, 2020; Fernandes et al., 2022).

Over the past several years, there has been notable progress in advancing a multitude of promising compounds, each possessing distinct mechanisms of action, into clinical phases of development. However, it is worth noting that strains of mycobacteria that are resistant of these compounds have already emerged (Fernandes et al., 2022). This underscores the urgency of identifying novel antitubercular strategies, particularly those active against infections caused by XDR-TB. Within this context, proteolysis-targeting chimeras (PROTACs) protein degradation approach have emerged as an innovative approach for the discovery of novel antimicrobials, as noted by Békés (2022).

Targeted Protein Degradation (TPD) approach has been extensively investigated in the realm of anticancer agents. PROTACs induce a ternary complex between a protein of interest (POI) and an E3 ligase, facilitating polyubiquitination and subsequent degradation of the POI (Békés et al., 2022). This procedure relies on the covalent attachment of ubiquitin molecules to specific lysine residues of the POI, which enables it to be recruited to, and degraded by, the proteosome (Sakamoto, 2001). The catalytic and event-driven mechanism of action of PROTACS provide the potential for the compounds to be used in lower doses than conventional drugs, which represents a pharmacological gain by reducing the adverse effects. Furthermore, PROTACs only need to attach to, and not modulate the targets to exerts its action. Thus, it expands the range of the druggable targets that can be explored, including non-enzymatic proteins. The engagement of two different macromolecules as long as the proper formation of a ternary complex between them improves the selectivity of these molecules, highlighting the advantages in using PROTACs (Pettersson, 2019; Sun, 2019; Burslem, 2020; Bekes, 2022; Liu, 2022; Sincere, 2023). Therefore, the applicability of the PROTAC technology in the field of antimicrobials emerge as a powerful strategy (Grohmann, 2023).

While the ubiquitin system is exclusive to eukaryotic cells, certain bacteria use a similar degradation system that relies on the recognition of phosphorylated arginine residues (pArg) by the ClpC:ClpP (ClpCP) protease, thereby leading to protein degradation (Trentini, 2016). This unique bacterial pathway hinges upon the critical role played by the ClpC subunit, particularly its ClpC_NTD_ protomer, in facilitating pArg recognition and the subsequent translocation of the target protein into the ClpP compartment for degradation (Wang, 2011).

Mycobacteria harbour an analogous protease system known as ClpC1P1P2, which plays a pivotal role in cellular survival, even within macrophages (Rengarajan, 2005). For mycobacteria, the caseinolytic protease (Clp) complexes is composed by ClpP1 and ClpP2 subunits, which associate with the unfoldase ClpC1 or ClpX to form the active protease complex (Taylor et al., 2023). Recently, Morreale and others demonstrated that PROTACs could similarly harness this mycobacterial protein degradation machinery to target neo-substrates in bacteria (Morreale, 2022) The researchers engineered a bifunctional molecule comprising a phosphorylated arginine moiety, at one end, which served to mimic the degradation tag, a linker, and a POI-specific binder at the other end. These innovative molecules were coined BacPROTACs (Morreale, 2022).

**FIGURE 1:**
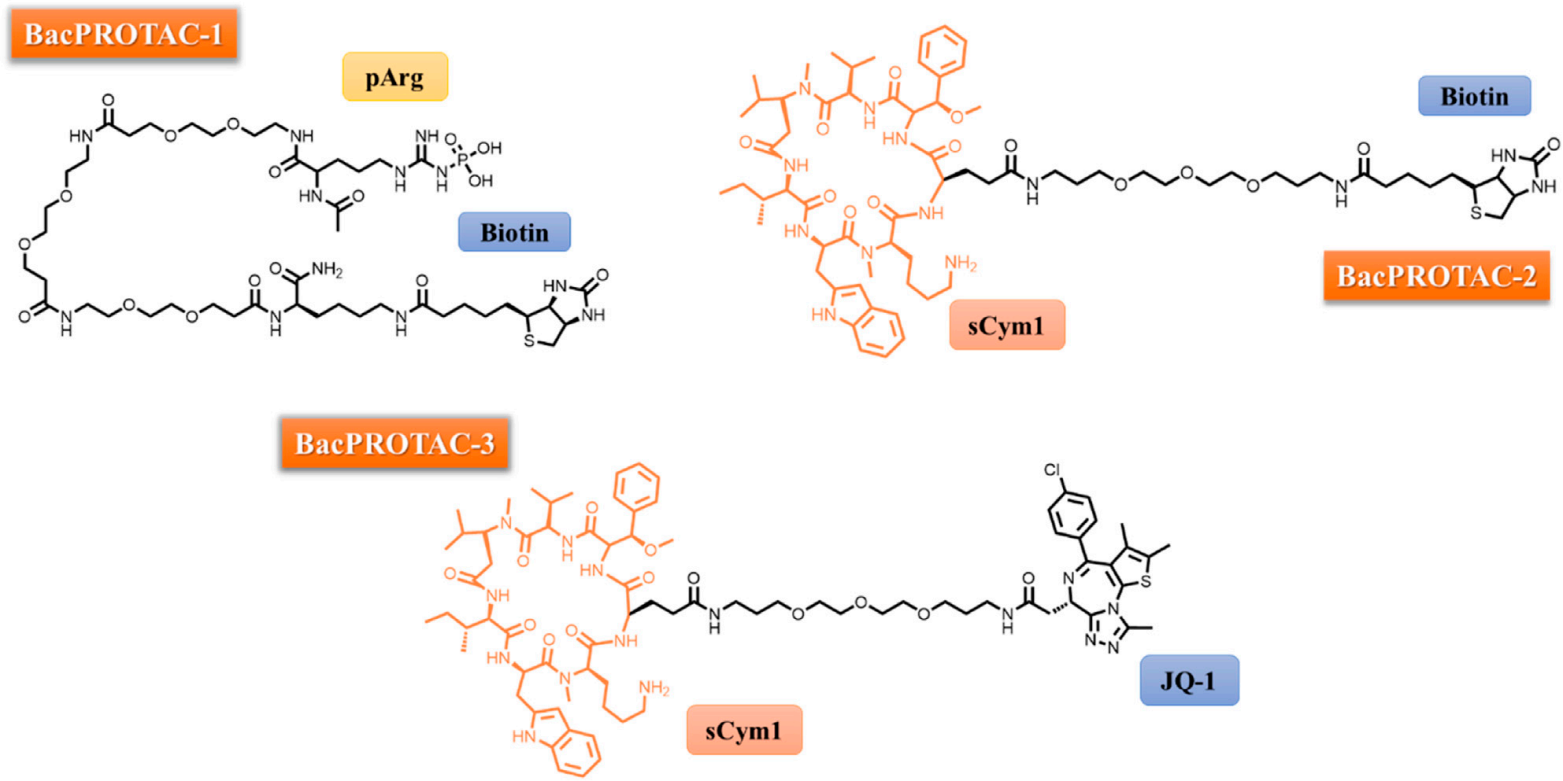
Chemical structure of BacPROTACs-1, -2 and -3.

In the initial phase of their study, the authors selected monomeric streptavidin (mSA) as their POI and proceeded with the synthesis of BacPROTAC-1 (Figure 1), incorporating biotin as a ligand for the POI. They employed isothermal titration calorimetry (ITC) to confirm that the compound maintained its ability to bind to both mSA and ClpC_NTD_ individually, with dissociation constants (Kd) of 3.9 and 2.8 mM, respectively. Additionally, analytical size-exclusion chromatography (SEC) demonstrated the formation of a stable ternary complex, underscoring the molecule’s capacity to simultaneously engage with the POI and Clp_NTD_. Subsequently, *in vitro* investigations involving the treatment of a reconstituted CplCP from *B. subtilis* with BacPROTAC-1led to the selective degradation of mSA, notably observable at a concentration of 100 µM. To further probe the substrate specificity of this innovative PROTAC, the authors cloned four fusion proteins in *Bacillus subtilis* (NdrI, TagD, NusA, and Kre) and subjected them to BacPROTAC-1 treatment. Significant differences in degradation efficiency were observed between the 4 substrates, with mSA-KRE degraded at as low as 1 μM, shedding light on the nuanced impact of structural variations within the substrate on the degradation pattern (Morreale, 2022).

When utilizing PROTAC technology, it is crucial to validate that observed outcomes are inherently linked to the activity of the designed molecule. To address this critical aspect, BacPROTAC-1 was meticulously applied to treat ClpCP in tandem with both pArg and biotin in isolation. This approach effectively resulted in the inhibition of mSA-Kre degradation, as the binding sites crucial for the formation of a ternary complex were preoccupied by these molecules. A parallel series of experiments yielded akin results when a non-phosphorylated arginine moiety was integrated into the BacPROTAC structure. Notably, the linker length did not exert a significant influence on the degradation process, which contrasts with the significant impact of linkers observed in other TPD studies (Troup et al., 2020; Bemis et al., 2021).

As part of the ongoing research, BacPROTAC-1 was subsequently assessed within the reconstituted ClpC1P1P2 complex derived from *Mycobacterium smegmatis*. This evaluation unveiled the molecule’s capacity for high-affinity binding to the ClpC1_NTD_, with a Kd of 0.69 mM. Importantly, this interaction facilitated the formation of a ternary complex involving mSA and ClpC1_NTD_, culminating in the selective degradation of mSA. These findings not only underscored the suitability of ClpC1P1P2 from mycobacteria for the advancement of PROTAC technology but also opened avenues for overcoming the limitations associated with the pharmacokinetics of pArg, as expounded by Schmidt in 2014. In response to this challenge, BacPROTAC-2 was designed, in which the pArg subunit was substituted with cyclomarin A (CymA), a cyclic peptide renowned for its robust binding affinity to ClpC1_NTD_ and enhanced permeability across the mycobacterial envelope (Schmitt, 2011a). It is noteworthy that ClpC1 serves as the target for an array of effectors, encompassing diverse natural products such as cyclomarin A, ecumicin, lassomycin, and rufomycin (Hoi, 2023). Cyclomarin and ecumicin exhibit comparable affinities for both ClpC2 and ClpC3, two small Clp proteins, and this interaction may lead to the upregulation of proteins (Hoi, 2023).

Subsequently, BacPROTAC-2 (Figure 1) was synthesized and its efficacy in degrading mSA was assessed. BacPROTAC-2 displayed a degradation pattern akin to that observed with pArg derivatives, underscoring its potency in TPD. To further establish the versatility of the mycobacterial protease in targeting various proteins, BacPROTAC-3 (Figure 1) was designed to incorporate JQ-1, a ligand for bromodomain-1 of BRDT (BRDT_BD1_), a human protein with no structural analogues in mycobacteria. When *M. smegmatis*, genetically modified to express BRDT_BD1_, was subjected to treatment with BacPROTAC-3 at a concentration of 10 μM, a substantial reduction of nearly 50% in target protein levels was observed. Notably, individual components of BacPROTAC-3, namely Cym and JQ-1, exhibited no discernible impact on the target protein levels and co-incubation of JQ-1 and BacPROTAC-3 competitively inhibited protein degradation. Furthermore, a new compound (BacPROTAC-3a) containing the distomer of JQ-1 showed no effect on the target protein levels, confirming that productive engagement of BRDT_BD1_ is required for the mechanisms of action of BacPROTAC-3 (Morreale, 2022).

CymA, a natural product known for its high binding affinity to ClpC1_NTD_, exerts a pronounced inhibitory effect on ClpC1 activity, an unfoldase that interacts with the ClpP1P2 protease to assemble a confined degradation chamber (Vasudevan et al., 2013). Phenotypic assays against *Mycobacterium tuberculosis* showed that CymA was active against several MDR-TB clinical isolates and hypoxic non replicating *bacillus*, without effects against Gram-negative and Gram-positive bacteria (Schimitt et al., 2011b). Using molecular modification approach, it was synthesized a cyclic peptide with a slightly simplified structure, named desoxycyclomarin C (dCym) (Junk et al., 2023).

Compound dCym, an analogue of CymA, has the capability to disrupt the proteome of mycobacteria, notably characterized by a 600-fold increase in ClpC2 levels (Hoi, 2023). ClpC2 is a protease protector striking similarity to the receptor domain of ClpC1. This similarity enables ClpC2 to bind to dCym, thereby preventing its interference with ClpC1, ultimately mitigating dCym’s toxicity by a factor of 4 in *M. smegmatis*. ClpC2, as well as the recent described ClpC3, serve as regulatory components within the Clp complexes, sharing the same ligand-binding site as observed in ClpC1. In doing so, they operate by engaging in competition for substrate binding (Hoi et al., 2023; Taylor et al., 2023). However, while ClpP2 is crucial for delivering substrates to the proteolytic complex, the proteolytic activity of ClpP1 alone is both necessary and sufficient for the degradation of at least some Clp substrates (D’Andrea et al., 2022).

**FIGURE 2:**
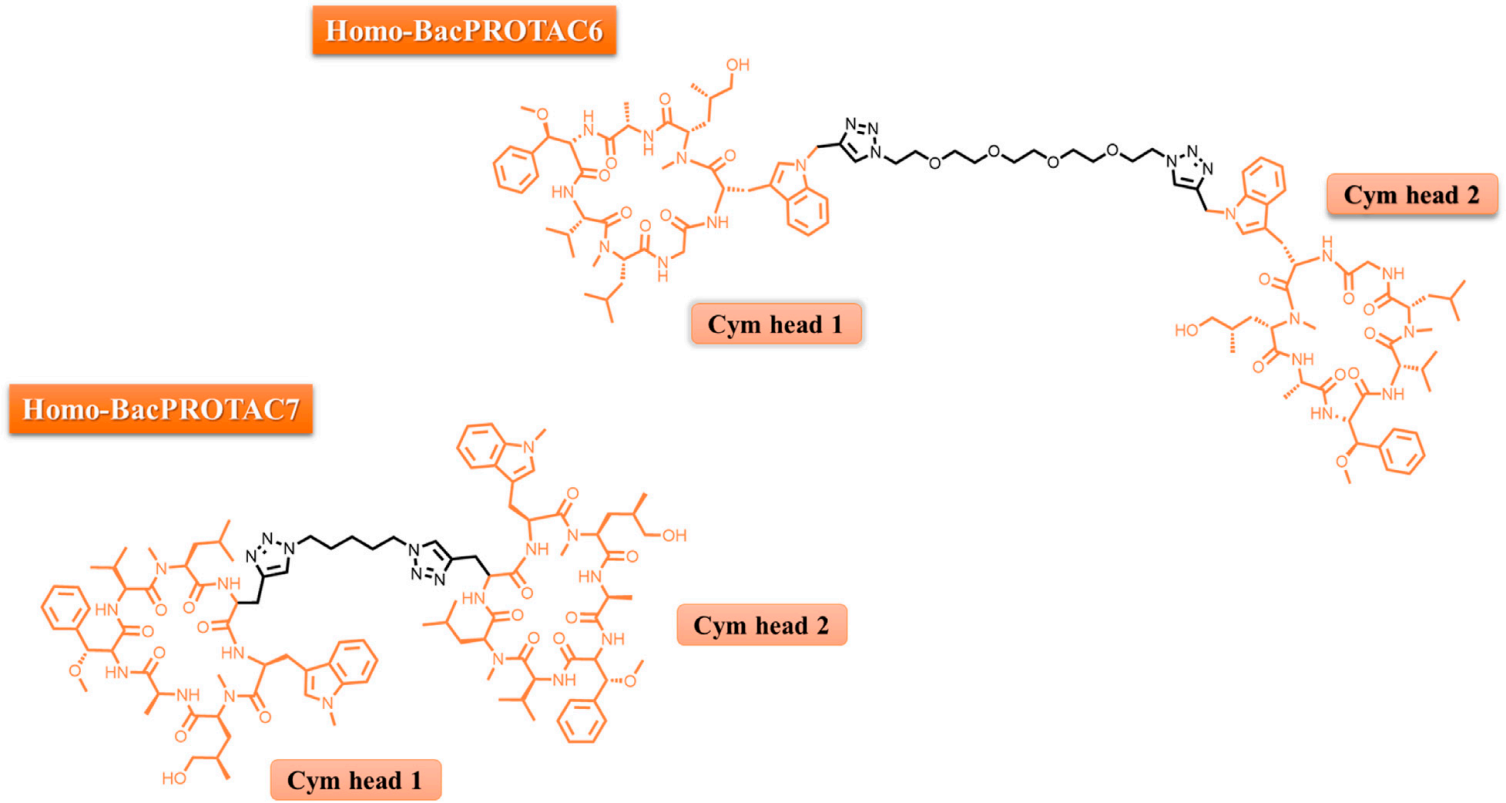
Chemical representation of HBPs 6 and 7.

To disrupt the “protective” effect against dCym and CymA mediated through the competitive binding to ClpC2/ClpC3, Hoi and others synthesized cyclic peptides dimers, which was named Homo-BacPROTACs (HBP). These HBP were synthesized to feature the dCym moiety at both ends connected by a different linker length (Figure 2). The goal was to simultaneously degrade both ClpC1, ClpC2 and the complex ClpC1P1P2. By using a *M. smegmatis* model system, it was found that these Homo-BacPROTACs were able to reduce the levels of ClpC1 and ClpC2 up to 40% and 45%–60%, respectively, in comparison with the monomeric dCym, after 24 h of treatment. The antimycobacterial effects was evaluated using *M. tuberculosis* H37Rv. In this phenotypic assay, the MIC_50_ values for HSP-6 and HSP-7 were 0.34 and 0.26 µM, respectively. In this experiment the MIC_50_ value for dCym was 39 µM. Both compounds were able to reduce ClpC1 and ClpC2 levels, and was active against dormant state of the *M. tuberculosis* (Hoi et al., 2023).

**FIGURE 3:**
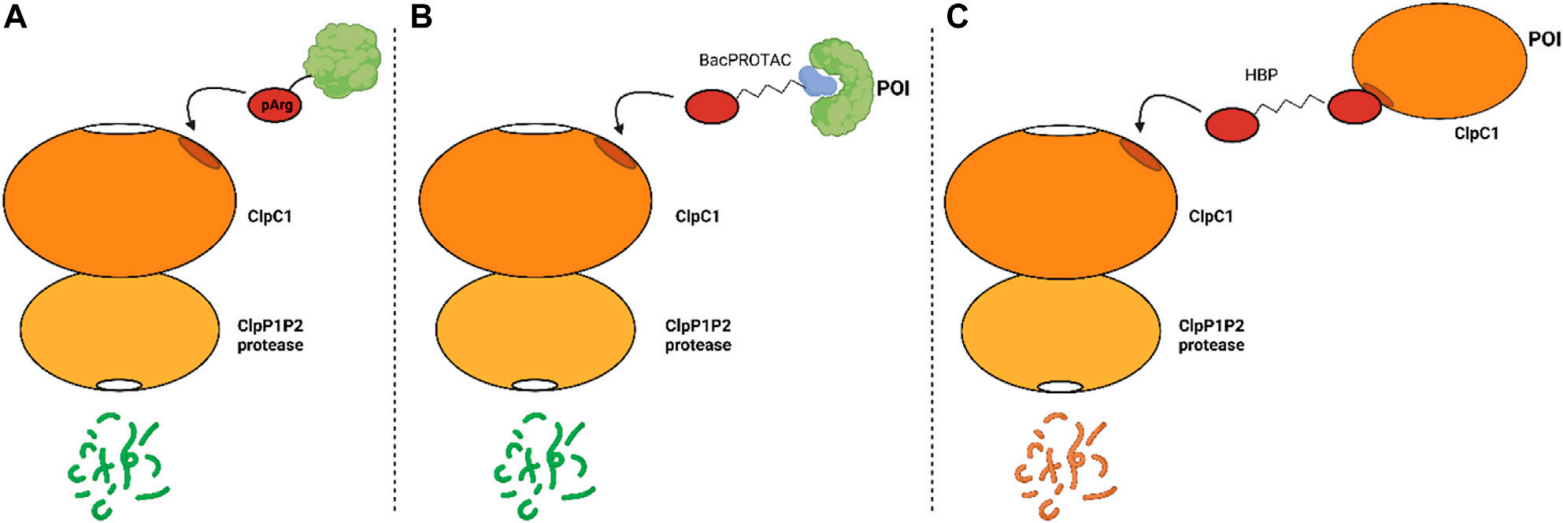
Mechanism of proteolysis mediated, or not, by synthetic compounds. **(A)** natural occurring protein degradation mediated by the recognition of pArg by ClpC1; **(B)** protein degradation induced by the BacPROTAC and **(C)** degradation of ClpC1 induced by HBPs.

Figure 3 presents a summary of the mechanism of the proteolytic machinery, as well as the BacPROTACs and HBPs.

Although advances have been achieved in the last years, the range of known small molecule ligands for the ClpP1P2, ClpC1, and ClpC2 complex remains relatively limited. Yang and others described that the anticancer cediranib, a potent inhibitor of the VEGF family receptor tyrosine kinases, is a potent ClpP1P2 inhibitor in *M. tuberculosis.* Cediranib was identified through a screening of a vast library of compounds. *In vitro*, this drug inhibited the complex ClpP1P2 with IC_50_ value of 2.8 µM and a selective index of 13.3 in comparison with human peptidase/protease. Through structural and mutational studies, it has been revealed that cediranib binds to MtbClpP1P2 by interacting with an allosteric pocket located in the equatorial handle domain of the MtbClpP1 subunit. Notably, phenotypic assays conducted with *M. tuberculosis* revealed MIC_50_ values against H37Ra and H37Rv of 14.1 and 28.2 µM, respectively. Minimal growth inhibition was noted in other species, including *E. coli*, *S. aureus*, and *P. aeruginosa* (Yang et al., 2023).

Another example of ClpP1P2 inhibitor is the proteasome inhibitor bortezomib. This drug was approved by US FDA for the treatment of multiple myeloma and mantle cell lymphoma, and it was identified in a fluorescence-based assay as a potent ClpP1P2 inhibitor. Whole-cell phenotypic assays carried out with *M. tuberculosis* H37Rv showed by using bortezomib MIC_50_ values of 0.8 µM (Moreira et al., 2015). To mitigate the effects on human proteasome, a series of peptide boronic compounds were described by Moreira and others. After a vast structure activity relationship (SAR) study, the researchers have found compounds that exhibited up to a 100-fold reduction in activity against the human proteasome yet retained both ClpP1P2 inhibition and effectiveness in inhibiting mycobacterial growth (Moreira et al., 2017). Therefore, while the human proteasome inhibitor bortezomib presents itself as a compelling scaffold for BacPROTAC design, it is imperative to eliminate its effects on the human proteasome and optimize interactions with ClpP1P2.

The outcomes stemming from the implementation of PROTAC technology in the quest for novel antimicrobials represent a beacon of hope, particularly in light of the escalating challenges posed by antibiotic resistance. The proliferation of antibiotic-resistant strains, including the MDR and XDR variants, as exemplified in the context of TB, has accentuated the urgency of innovative approaches. PROTAC technology, by enable the design of degraders, now stands as a formidable driver in expanding the realm of druggable targets. Such approach is a promising tool by targeting biomolecules that need not harbour traditional active sites and exhibits several advantages, including: reduction of pressure to evolve resistance; improve the selectivity, as require engagement of two distinct targets and formation of ternary complexes; BacPROTACs are catalytic and event driven, which enables lower drug concentrations that could improve the therapeutic index of a drug, and by reducing side effects; BacPROTACs only require to bind (not modulate the function) of their target, which could increase the proportion of the bacterial proteome that can be pharmacologically modulated. Thus, these adaptable degraders can be tailored for a diverse range of targets, including structural proteins and protein aggregates, thereby transcending the conventional confines of target selectivity (Bekes, 2022). In essence, this emergent avenue furnishes the scientific community with an ever-expanding arsenal of tools to be used to overcome the problems we are facing nowadays in the drug discovery of antimicrobials.

## Data Availability

The original contributions presented in the study are included in the article/supplementary material, further inquiries can be directed to the corresponding author.
